# The impact of video gaming on cognitive functioning of people with schizophrenia (GAME-S): study protocol of a randomised controlled trial

**DOI:** 10.1186/s12888-020-03031-y

**Published:** 2021-01-18

**Authors:** Maritta Välimäki, Min Yang, Yuen Ting Joyce Lam, Tella Lantta, Matias Palva, Satu Palva, Benjamin Yee, Siu Hung Yip, Kin-sun Dan Yu, Hing Chiu Charles Chang, Po Yee Ivy Cheng, Daniel Bressington

**Affiliations:** 1 Xiangya Nursing School, Central South University, 172 Tongzipo Road, Changsha, 410013 Hunan China; 2 School of Nursing, The Hong Kong Polytechnic University, Hung Hom, Kowloon, Hong Kong, Hong Kong SAR; 3 Department of Nursing Science, Faculty of Medicine, University of Turku, 20014 Turku, Finland; 4 West China School of Public Health, Sichuan University, Chengdu, China; 5 Faculty of Health, Art and Design, Swinburne University of Technology, Melbourne, Victoria 3122 Australia; 6 Neuroscience Center, University of Helsinki, Helsinki, Finland; 7 Department of Rehabilitation Sciences, Hung Hom, Kowloon, The Hong Kong Polytechnic University, Hong Kong, Hong Kong SAR; 8 Department of Psychiatry, Kowloon Hospital, Hong Kong, Hong Kong SAR; 9 The Mental Health Association of Hong Kong, 2 Kung Lok Road, Hong Kong, Hong Kong SAR; 10 Department of Diagnostic Radiology, The Hong Kong Jockey Club for Interdisciplinary Research, The University of Hong Kong, 5 Sassoon Road, Hong Kong, Hong Kong SAR; 11 Department of Psychiatry, Community Psychiatry, Pamela Youde Nethersole Eastern Hospital, Hong Kong, Hong Kong SAR; 12 College of Nursing and Midwifery, Charles Darwin University, Darwin, Australia

**Keywords:** Gaming, Randomised controlled trial, Schizophrenia, Effectiveness

## Abstract

**Background:**

Video gaming is a promising intervention for cognitive and social impairment in patients with schizophrenia. A number of gaming interventions have been evaluated in small-scale studies with various patient groups, but studies on patients with schizophrenia remain scarce and rarely include the evaluation of both clinical and neurocognitive outcomes. In this study, we will test the effectiveness of two interventions with gaming elements to improve cognitive and clinical outcomes among persons with schizophrenia.

**Methods:**

The participants will be recruited from different outpatient units (e.g., outpatient psychiatric units, day hospitals, residential care homes). The controlled clinical trial will follow a three-arm parallel-group design: 1) cognitive training (experimental group, CogniFit), 2) entertainment gaming (active control group, SIMS 4), and 3) treatment as usual. The primary outcomes are working memory function at 3-month and 6-month follow-ups. The secondary outcomes are patients’ other cognitive and social functioning, the ability to experience pleasure, self-efficacy, and negative symptoms at 3-month and 6-month follow-ups. We will also test the effectiveness of gaming interventions on neurocognitive outcomes (EEG and 3 T MRI plus rs-fMRI) at a 3-month follow-up as an additional secondary outcome. Data will be collected in outpatient psychiatric services in Hong Kong. Participants will have a formal diagnosis of schizophrenia and be between 18 and 60 years old. We aim to have a total of 234 participants, randomly allocated to the three arms. A sub-sample of patients (*N* = 150) will be recruited to undergo an EEG. For neuroimaging assessment, patients will be randomly allocated to a subset of patients (*N*=126). We will estimate the efficacy of the interventions on the primary and secondary outcomes based on the intention-to-treat principle. Behavioural and EEG data will be analysed separately.

**Discussion:**

The study will characterise benefits of gaming on patients’ health and well-being, and contribute towards the development of new treatment approaches for patients with schizophrenia.

**Trial registration:**

ClinicalTrials.gov NCT03133143. Registered on April 28, 2017.

## Background

More than 21 million people worldwide suffer from schizophrenia [1]. Schizophrenia is associated with severe disabilities in cognition and global functioning [2, 3]. Deficits in attention, executive function and working memory are the core challenges for this patient group [4, 5]. Having a working memory is a crucial part of daily functioning and holding and manipulating information [6]. Poor working memory is associated with poor functional outcome [4] and limits for social relationships [7]. Disabling cognitive symptoms in schizophrenia are associated with prefrontal dysfunction [8].

Cognitive deficits can be improved with specialised cognitive training programmes. For example, remediation therapy is designed to improve attention, memory, and problem solving [9]. Various types of computer-based applications for cognitive remediation have also been established, as well as serious games, which are specialised for training cognitive deficits. Fisher et al. tested a set of computerised exercises in patients with schizophrenia and showed that those who made the most progress in the basic psychophysical auditory exercise also exhibited the most improvement in verbal working memory and global cognition [10]. More recently, video gaming in general (on a computer, console, online platform, or mobile device) and sole entertainment games (action, sport, role-playing, adventure, fighting, racing, family entertainment, casual games) [11] have opened up an avenue for new remedial interventions targeting attention, problem-solving, emotional expression, and socialization [12]. This approach is potentially more effective than direct instructional methods, such as coaching, which yields limited benefits for patients with schizophrenia [13]. Kühn et al., for example, reported that video gaming increases grey matter volume in the brains of healthy adults, and they recommended gaming for patients with schizophrenia [14].

Functional magnetic resonance imaging (fMRI) studies have recently been used in working memory tests for patients with schizophrenia [15]. Some studies have reported increased activation of the dorsolateral prefrontal cortex (DLPFC) in patients with schizophrenia during their working memory performance [16]. Imaging methods have also been used to assess the effectiveness of gaming interventions for neurocognitive outcomes. One fMRI study suggested that persons who have played a shooter game improved their attentional resources more efficiently than non-gamers [17]. A systematic review further suggests action video games as a promising method in mental health treatment, as asserting that specific types of video games can alter brain structure or improve certain aspects of cognitive functioning [18]. Video gaming may be particularly useful for persons who have difficulties in treatment compliance or engagement because gaming itself may offer positive experiences and emotions [19, 20], a sense of self-efficacy, relatedness, and social interaction [21, 22]. These effects are expected to benefit patients with schizophrenia [2–5]. Further, video gaming is cost effective and allows personalised targeting of specific brain abnormalities by ameliorating the dysfunction in specific brain circuits and normalizing network dynamics. When suitably scaled, gaming may be highly effective in countering the impact of risk factors associated with prodromal states of various mental disorders [14, 22]. This suggestion is in keeping with speculation that some commercially available video games may already have the potential to cause changes in human behaviour [23]. However, the influences of video gaming on brain function and structures, in relation to its potential benefits for cognitive functioning, social relationships [18], pleasure and self-efficacy, remains poorly characterised [13]. In addition, the most useful setting for the implementation of gaming interventions is still unclear [24].

We hypothesise that playing video games [13] may alter brain function, cognition and behaviour [23] for patients with schizophrenia [25]. Benefits for prospective memory impairments and emotion-behaviour decoupling related hedonia have been demonstrated in schizophrenia patients [26]. One neuroimaging study outside of the field of mental health shows that both early stimulus processing and late retention period activities associated with video gaming could improve visual working memory function, and suggests that video gaming might be suitable for patients with schizophrenia [27]. Indeed, there is some evidence that entertainment games can be useful in mental health in, for example, improving moods or stress levels [28, 29]. However, these are studies with small sample sizes and narrow scopes in age groups [30].

Based on the current role of video gaming [10], its rapid expansion in the market, and its promises in treatment for persons with schizophrenia, it is important to conduct a larger-scale investigation to fully characterise its potential clinical profile and efficacy [30]. Here, we will test the effectiveness of gaming embedded within two interventions in a longitudinal study on relevant cognitive and clinical outcomes, as well as EEG and neuroimaging assessments (structural MRI, resting-state fMRI, and diffusion tensor imaging) as a form of brain physiological readout.

## Methods

### Objectives

This study aims to compare the effectiveness of cognitive training using computerized gaming exercises to that of entertainment video gaming and a non-gaming, passive control group, looking at a range of outcomes. The primary objective is to test the effects of the interventions on patients’ cognition, especially on working memory at 3-month and 6-month follow-ups. The secondary objectives are to test the effects of the interventions on patients’ other cognitive and social functioning, the ability to experience pleasure, self-efficacy, and negative symptoms at 3-month and 6-month follow-ups. Additional secondary outcomes are EEG brain activity and structure related to working memory function at a 3-month follow-up.

### The hypotheses

Primary hypothesis:

1. Cognitive training with computerised exercises is more effective than entertainment video gaming or the non-gaming control in improving patients’ working memory at a 3-month and/or 6-month follow-up.

Secondary hypotheses:

2. Cognitive training with computerised exercises is more effective than entertainment video gaming and the non-gaming control in improving patients’ cognitive and social functioning, experience of pleasure, and self-efficacy at a 3-month and/or 6-month follow-up.
3. Cognitive training with computerised exercises is more effective than entertainment video gaming and the non-gaming control in improving negative symptoms in schizophrenia at a 3-month and/or 6-month follow-up.
4. Cognitive training with computerised exercises is more effective than entertainment video gaming and the non-gaming control in improving neurocognitive outcomes (EEG signals, brain activity and structure) at a 3-month follow-up.

### Trial design

The effectiveness of the intervention will be assessed using a controlled clinical trial with a pragmatic, three-arm parallel-group design. The three-arm design is chosen to explicitly test the superiority of the experimental cognitive training with computerised exercises (CogniFit) compared to the active control (entertainment games) and treatment as usual (non-gaming passive control) [31]. Since the objective of the study is to test the efficacy of the type of gaming (cognitive training with computerised exercises vs. entertainment gaming), having a three-arm trial is necessary to assess the effects of the gaming types separately.

To assess fidelity, we will use gaming diaries to verify whether the interventions have been delivered as intended (intervention fidelity) and confirm patient gaming activity (gaming frequency [number of gaming sessions per week], the length of each session [minutes], number of drop-outs). We will also analyse any information on the perceived strengths and limitations of interventions written in the patients’ diaries.

### Study setting and participant characteristics

The data collection, subject recruitment and gaming will be carried out in outpatient services (e.g., outpatient psychiatric units, day hospitals, residential care homes). Specific inclusion criteria mandate that patients should have a formal diagnosis of schizophrenia spectrum disorder (Diagnostic and Statistical Manual of Mental Disorders, fifth edition, DSM-5), and be between 18 and 60 years old. We will intentionally recruit people who are non-active, less-experienced gamers [19]. Participants will be required to be able to speak Cantonese, be able to participate based on their own free will and have the ability to provide written informed consent. Participants should have a cognitive status deemed suitable for study participation based on ‘the judgement standard’, i.e., be approved by the staff responsible of the treatment based on their clinical expertise.

The exclusion criteria are: 1) meeting the diagnostic criteria for a current major depressive, manic or hypomanic episode (DSM-5), or mental retardation, 2) having severe visual impairment, 3) being an active gamer (i.e. gaming > 5 h/week [17]), 4) displaying a lack of ability to decide one’s own participation, 5) substance abuse (other than nicotine dependence), 6) head injury, hemiplegia, or other neurological disorder, 7) electroconvulsive therapy (ECT) in the past six months, 8) having a lack of Magnetic Resonance Imaging (MRI) compatibility (e.g., cardiac pacemakers, metallic implants, restless behaviour, claustrophobia), and 9) pregnancy.

### Power analysis and sample size

We have calculated the sample size based on the primary outcome as follows: (1) the number of actual pairwise tests to be made for the efficacy of the primary outcome and (2) the two-level modelling approach in the final data analysis, in which the type I error has been adjusted. The statistical efficiency will be ensured using the multivariate analysis of variance (MANOVA) method for multiple group comparison. First, given that video gaming is a fairly novel strategy, we will base the sample size calculation (a priori) [32] on a cognitive-efficacy meta-analysis for patients with schizophrenia [33] showing an overall effect size (ES, Cohen’s *d*) of 0.58 on verbal working memory (a primary outcome). Based on our hypothesis, the primary endpoints will be the effects on verbal working memory after 3 and 6 months of cognitive training group (CogniFit), compared with the other two groups: cognitive training vs. entertainment gaming, cognitive training vs. non-gaming control group, and entertainment gaming vs. non-gaming control group. Four pairwise interactions between the contrast of the two comparisons and the two time points will be tested. For multiple comparison tests of four, for a type I error level of 5% (two-sided), an adjusted significant level should be α/2 = (1- (1–0.05)4) /2 = 0.01274 /2 = 0.0064, and the corresponding z score for a one-tailed test should be 2.49. Given the effect size 0.58, assuming equal sample size of the three groups, with a statistical power of 0.8 and α= 0.01274*,* we will require at least 198 subjects (66 per group at follow-up phase) by applying the equation, 2(Z1-β + Z1-α/2)2 / ES2. According to a meta-analysis [33], the total sample size in previous cognitive training studies was around 50 (range 10–138).

Using evidence-based rationale for patient flow in this study, we can assume that about 60% of patients screened will not be eligible for the study, due to age or lack of capacity to participate in the study [34]. Based on existing literature, we anticipate that about 45% of patients with schizophrenia will refuse to participate in the RCT studies [33], about 60% of patients screened will not be eligible for the study, and around 16% will drop out during the course of intervention [35]. Thus, we need a total of 1073 subjects to be approached and 237 participants to be randomly allocated to three study groups (79 patients/group) to ensure a final sample of 198 participants in the follow-ups (66 patients/group). These numbers are realistic given that the total number of patients with schizophrenia in our study sites is about 5500, and the total number of schizophrenia patients in Hong Kong is 40,000.

**Fig. 1:**
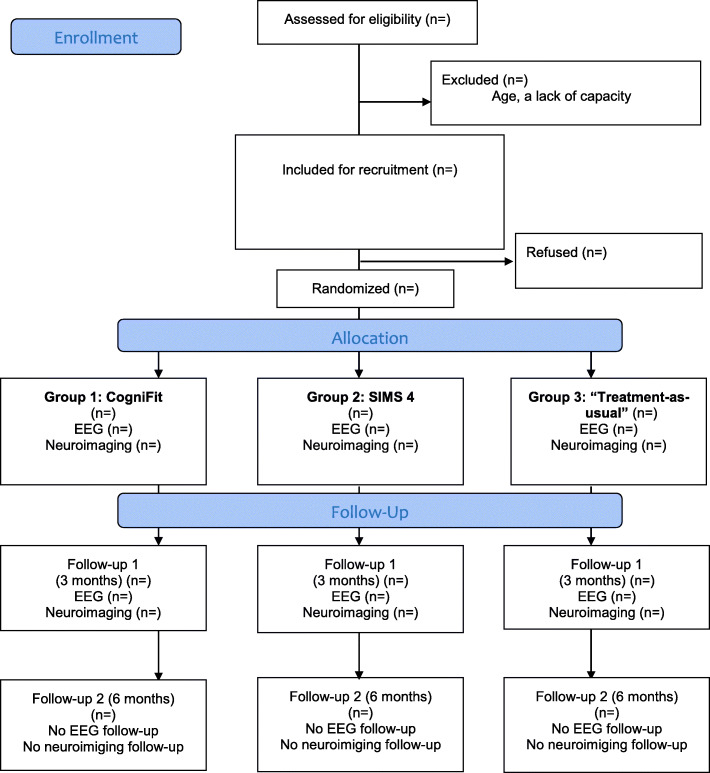
Flow chart of the GAME-S study

For the EEG analysis, a sub-sample of 50 patients in each group will be recruited (*N* = 150). For neuroimaging assessment using 3 T MRI and rs-fMRI, a sub-sample of 126 participants from our total sample (*N* = 198) will need (63%) to be randomised at baseline. Randomisation will be based on a list of computer-generated random numbers. We assume that 30% will drop-out between baseline and the 3-month follow-up assessment, which leaves us with 29 patients in each group (a total of 87 participants at baseline). The sample size will be appropriate for our neuroimaging assessment; the average number of participants in RCT studies assessing patient cognition [33] or changes in brain structure has been about 20 [14]. The flow-chart of the study is presented in Fig. 1.

### Recruitment

For privacy purposes, the medical records in each service organisation will be screened by the clinical staff to determine the eligibility of patients to participate in the study. To ensure that patients have the capacity to make the decision to participate or not, an extended informed consent process will be used [36]. Eligible participants will first receive a short leaflet about the study from the staff or during a short information session organised for the participants to consider their availability. If an eligible patient shows interest, more detailed written and oral information will be given. It will be ensured that a patient understands the information and they will have the opportunity to ask any questions. If a patient is willing to participate in the study, they will sign an informed consent form. To ensure accuracy of the written information to be provided to subjects, its content will be revised whenever new information relevant to the subject’s consent becomes available. Prior to participation in the trial, the study participants will receive a copy of the signed and dated written informed consent form and any other written information provided to the subjects. If any updates occur, the participant will receive a copy of the signed and dated consent form updates and a copy of any amendments to the written information provided to subjects during their participation in the trial.

After signing the informed consent form, background information of each respondent, their Intelligence Quotient (IQ, if available in medical records) and Mini Mental State Examination (MMSE) will be collected [37]. Their medication dosages (chlorpromazine equivalence) will also be collected as medications currently used in schizophrenia may affect the response to cognitive training strategies [9, 37]. A simple practical test to assess the participants’ computer skills will be carried out (starting up a computer, using a mouse). If any doubts of computer use arise, our intervention is long enough (12 weeks) for participants to learn basic computer and gaming skills. The recruitment will end as soon as the planned total numbers of participants are reached for the analysis.

### Randomisation, allocation concealment and blinding (masking)

After baseline data collection, the trial manager will be notified of new participants. Each participant will be allocated to one of the three arms based on a list of computer-generated random numbers provided by an external clinical trial randomisation service (blocks of 6 consecutive patients, a 1:1:1 ratio). Allocation will be masked to the trial statistician, but it cannot be masked to the research assistants (RAs) who will supervise the gaming sessions and collect follow-up outcome data, or from the study unit staff who will work with the patients. Furthermore, a sub-sample of 150 participants will be recruited for EEG analysis, and 126 participants from our total sample will be randomly selected and assigned to neuroimaging assessment at baseline (fMRI). Again, the participants for neuroimaging assessment will be allocated based on a separate list of computer-generated random numbers provided by an external clinical trial randomisation service (blocks of 6 consecutive patients, a 1:1:1 ratio). The trial manager will inform the RAs the identities of participants allocated to fMRI assessment.

### Interventions

Participants will be allocated into three groups: 1) cognitive training with computerised exercises (G1, CogniFit, *N*=78), 2) entertainment video gaming (G2, Entertainment Gaming, *N*=78), and 3) treatment as usual (*N*=78). CogniFit and entertainment games (SIMS 4) represent two distinct gaming genres: CogniFit is a cognitive training package with computerised exercises aiming to improve cognitive abilities based on a personalised brain training regimen. SIMS 4 is a pure entertainment game without any known health impact. The administration of both interventions will include 60 pre-scheduled gaming sessions [38] at the study settings as part of the normal day programme, lasting from 45 to 60 min each, at least 5 days/week [17], 60 h in total. The gaming schedule can also be tailored based on the participants’ needs (working, studying, family issues) as long as the gaming hours are in line with the total hours planned. Individual gaming sessions on separate computers will be conducted in small groups (about 3–6 per group) and closely monitored by the trained research assistant. Monitoring of gaming sessions is crucial to ensure intervention fidelity. This was exemplified in our feasibility study, which showed that, out of all possible 60 pre-scheduled sessions, 97% of sessions were realised as planned, and all participants enjoyed gaming. Gaming hours (diaries, automatic gaming logs) for each gaming session will be recorded to provide evidence of the intervention fidelity; a minimum of 50 gaming hours will be acquired to ensure observable neuroplasticity after gaming [19]. To report any negative side effects of gaming (e.g., tiredness of eyes), feedback and any concerns will be reported in a gaming diary after each gaming session [39].

#### Cognitive training with computerised exercises (an experimental intervention group, CogniFit)

The supposed mechanism of CogniFit is derived from evidence that computerised exercises focusing on auditory and verbal processing are likely to yield improved verbal learning and memory [12], and activate the brain reward system that drives brain plasticity in adults with schizophrenia [40, 41]. Exercises include the elements needed for an effective cognitive intervention: a large number of specific repetitive learning trials driven by maximally enduring, neurologically reliable cognitive gains, and individually adapted content based on each learner’s needs [12]. To match the training regimen for each participant’s unique cognitive needs (deficits), a short assessment using CogniFit exercises will be done at the beginning of the intervention. During each training session, the participants will be instructed to play memory games and at least one exercise from each of the three categories: memory, spatial perception, and mental planning. Otherwise, they will be free to choose which exercises they wish to play.

During the first gaming session, a game will be introduced to the participant, and its use will be tested together with an RA. The participant’s ability to play digital games will be explored to ensure that they have the basic gaming skills required for active gaming (how to start the computer, how to play the game, how to change game options, and so on). During each gaming session, an RA will be available for gaming support. The RA will also provide assistance, if required, based on each gamer’s individual needs.

#### Entertainment video gaming (an active control group)

The video game offers entertainment without any known cognitive or health-related outcome. We will use SIMS 4, which includes a variety of personalities portrayed as cartoon characters that be customised based on gamers’ needs and wishes. For example, a gamer can change the clothing of characters, develop their stories, build homes, have the characters travel and build relationships between characters. As in the intervention group, an RA will be available during each gaming session to guide and support gaming skills and initiatives.

#### Treatment as usual (a passive control group)

No specific gaming intervention will be offered to those who receive treatment as usual. To minimise the risk of intervention contamination, the participants will be encouraged not to play video games during the study period and will be asked to report any video gaming with which they may have engaged.

### Outcome evaluation (primary and secondary outcomes)

#### Primary outcome

Verbal working memory will be measured with the Letter-Number-Sequencing task (Letter-Number-Span-Test, LNST) from WMS III (Wechsler Memory Scale, the 3rd Edition, simplified/traditional Chinese versions). The instrument includes an NIMH-MATRICS (US National of Institute of Mental Health, the Measurement and Treatment Research to Improve Cognition in Schizophrenia) battery [42].

#### Secondary outcomes

##### Cognitive functioning

A battery of cognitive tests will be used to measure the processing speed [Trail Making Test, TMT, A [42]], attention and vigilance [Sustained Attention to Response Task, SART], visuospatial working memory [Spatial Span from WMS III [42]], and reasoning and problem solving [Wisconsin Card Sorting Test [42]]. The instruments to be used are well tested, reliable, recommended, and available in simplified/traditional Chinese, either in paper or digital format [42].

##### Social functioning

A Chinese version of the Brief Social Phobia Scale, BSPS, will be used for assessing the severity of and treatment response to social phobia [43]. The Cronbach’s alpha for the Chinese version is 0.88, which states a high level of internal consistency [43].

##### Experience of pleasure

The Temporal Experience of Pleasure Scale (TEPS, Chinese version) covers anticipatory and consummatory components (Cronbach’s alpha=0.83; test-retest reliability r=0.79) [44].

##### Self-efficacy

The Chinese version of the General Self-Efficacy Scale, GSE, a self-reporting measurement of self-efficacy with good internal consistency (Cronbach’s alpha = 0.91), will be used [45].

##### Negative symptoms of schizophrenia

The Chinese version of the Clinical Assessment Interview for Negative Symptoms (CAINS) [46] will be administered by qualified psychiatrists who have received proper training for this assessment tool. The item-total score correlation will also be presented for each item, and can range from 0.58 to 0.81. The internal consistency (Cronbach’s alpha) for the scale is high (0.91) [46].

##### Other

Depressive symptoms will be measured with the Calgary Depression Scale for Schizophrenia (CDS-C) [47], an observer-rated Likert-scale to measure depressive symptoms (Cronbach’s alpha=0.80; inter-rater reliability Kappa coefficient > 0.79; test-retest reliability r=0.927), the Simpson-Angus Rating Scale (SAS) [48], the Barnes Akathisia Rating Scale (BARS), and the Abnormal Involuntary Movement Scale (AIMS). Participants’ IQs will be collected from medical records (if available) and the Mini-MMSE will be used to verify each participant’s capacity to participate in the study. If, at baseline, it is obvious that the participant suffers from a condition that will interfere with participation in the study (IQ, MMSE), the participant will be excluded from the study. Permission to use each instrument has been obtained and license costs have been paid as necessary.

#### Neurocognition

##### Electroencephalography (EEG)

The impact of gaming on brain functional networks and changes in neuronal dynamics can be revealed by an EEG during the execution of a visual working memory task. Resting-state EEG will be collected to evaluate changes in spontaneous activities in the brain for comparison with the resting-state MRI. The EEG has been extensively utilised to assess the effectiveness of daily cognitive exercises to improve sensory information processing, from attention to working memory processes, in people with schizophrenia. Its superior temporal resolution is most apt in identifying relevant markers in neuronal network dynamics that underlie the observed improvement, that might help in further identifying subsets of patients most likely to benefit from cognitive exercises [49].

##### Resting-state fMRI

In resting-state functional (rsfMRI) and structural MRIs, the most often reported neurocognition measurement for regions includes that of the nucleus accumbens, amygdala, striatum, anterior cingulate cortex, orbitofrontal cortex, and dorsolateral prefrontal cortex [50], the dorsal lateral prefrontal cortex and the anterior cingulate Testgyrus [51]. During the rsfMRI, the subject will not perform any tasks while being scanned. The rsfMRI is based on the principle that, even when the brain is at rest, there are still low fluctuations of neural activity. While the resting-state neural activity is at a much lower rate than when the brain is active, it can be used as a biomarker to study the underlying neuronal networks.

Comparing pre- and post-training records will reveal the impact of cognitive training with computerised exercises on the neuronal networks underlying the physiological basis of working memory ability. We will assess how the training influences inter-areal functional and structural (measured with MRI/Diffusion tensor imaging [DTI]) connectivity and the quantitative indices of cortical criticality and excitation/inhibition balance.

### Data collection and follow-up

To avoid burdening the patients, data will be collected in separate phases (see Table 1):

1. A preparatory data collection (estimated 45 min) will be collected only once, at the beginning of the study. It aims to collate the background information of the participants to determine their capacity for informed consent and any other potential factors that might restrict participation in the intervention (background information, IQ, medication dosage [based on medical records], MMSE, CDS-C, SAS, BARS, AIMS).

2) For the primary (verbal working memory) and secondary outcomes (estimated 75 min) (cognitive and social functioning, experience of pleasure, self-efficacy, negative symptoms), the data will be collected at three points in time: at baseline, after the intervention (3-month follow up) and at a six-month follow-up (6 months after baseline).

3) The EEG measures taken during the working memory test will be collected at baseline and 3 months after baseline only (EEG 1 h; rsfMRI and structural MRI, 45 min).

**Table 1:** Instruments and study stages

TIME POINTS Instruments	Eligibility screening	Preparatory data	Baseline	Allocation	Intervention	3 month follow-up	6 month follow-up
Inclusion/exclusion criteria	**X**						
Background information		**X**					
IQ (from medical records if available)		**X**					
Current medication dosage		**X**					
Computer use		**X**					
MMSE		**X**					
Calgary Depression Scale for Schizophrenia, CDS-C		**X**					
Simpson-Angus Rating Scale, SAS		**X**					
Barnes Akathisia Rating Scale, BARS		**X**					
Abnormal Movement Involuntary Scale, AIMS		**X**					
**Primary and secondary outcomes**							
Letter-Number-Span-Test, LNST			**X**			**X**	**X**
Trail Making Test, TMT			**X**			**X**	**X**
Sustained Attention to Response Task, SART			**X**			**X**	**X**
Spatial Span			**X**			**X**	**X**
Wisconsin Card Sorting			**X**			**X**	**X**
Brief Social Phobia Scale, BSPS			**X**			**X**	**X**
Temporal Experience of Pleasure Scale, TEPS			**X**			**X**	**X**
General Self-efficacy Scale, GSE			**X**			**X**	**X**
Clinical Assessment for Negative Symptoms, CAINS			**X**			**X**	**X**
EEG^a^			**X**			**X**	
MRI^b^			**X**			**X**	
ACP^c^				**X**			
**Study groups**							
Cognitive training with computerized exercises (experiment group)					X		
Entertainment video gaming (active control group)					X		
TAU (passive control group)							

^a^Convenience sample; ^b^Randomised subsample; ^c^ACP test planned but need to discontinue due to technological problems of the measure

First, for the primary and secondary outcomes (cognitive and social functioning, experience of pleasure, self-efficacy, negative symptoms), the data will be collected in paper format or digital format [5] by a trained psychologist or RA.

Second, the EEG will be recorded during resting state and a multi-objects visual working memory task (VWM). The task, modified from our prior studies [52], will be performed so that the neuronal mechanisms limiting VWM performance and capacity can be identified during the memory retention period. Subjects will attend either hemifield, depending on the pre-stimulus cue, and memorise a visual display containing one to six objects. After the retention period, a second stimulus will appear, and the subject will answer whether the stimulus presented in the second display matches part of the first display or not. We will correlate both local and large-scale neuronal dynamics with the task performance, both in pre-training and post-training neuroimaging sessions.

Third, fMRI scans will be acquired using a Philips 3 T MRI scanner. During scanning, a painless procedure, patients will rest on a table, which will be slid into a large tunnel-shaped scanner. As there will be a constant drumming noise during scanning, earplugs or earphones will be provided. During the examination, the patient will be able to freely communicate with staff via an intercom. Resting-state fMRI data will be acquired using T2-weighted echo planar pulse sequence imaging (300 whole-brain volumes were collected with slice thickness=4 mm, TE=30 ms, TR= 2000 ms, flip angle=90 degrees, spatial resolution 3x3x4 mm isotropic voxels, transverse orientation, 32 slices fully covering the cerebral cortex and the cerebellum, acquisition time = 10 min). Structural MRIs will be acquired with the MP-RAGE T1 sequence. A DTI of the axonal tracts in cerebral white matter will be performed with a protocol validated in the Human Connectome Project. We will acquire a 15-min session of resting-state data to obtain high-quality measurements of intrinsic functional connectivity of BOLD signals (fMRI) [53] and critical dynamics (fMRI) [54].

### Data analysis

#### Cognitive and clinical outcomes

The demographic information and outcome measurements of the participants in the three study groups will be compared for similarities at baseline. Based on the types of variables, Chi Square tests and analyses of variance (ANOVA) will be used for categorical and continuous variables, respectively. Variables found significantly different between study groups will be controlled in the main analysis as covariates. The normality assumption of the outcome scores will be tested (Kolmogorov-Simonov tests). Outcome measures of non-normal distribution will be transformed to normal in the main analysis.

In the main analysis, we will estimate and test efficacy of interventions on the primary and secondary outcomes. Based on the intention-to-treat principle, we will first assess intervention effects on the primary outcome, verbal working memory, by means of multilevel modelling analysis. A two-level model can be built with repeated time points at level 1, and patients at level 2. Two indicator variables, G2 and G3, will be included in the fixed part of the model to estimate and test mean differences between the cognitive training group with computerised exercises (G1) and the entertainment gaming group (G2), as well as between G1 and the non-gaming control group (G3). The baseline difference between G2 and G3 can be tested using a Ward test, based on the regression coefficient estimates of the two indicators. For changes in the outcome over time, two more indicators, variables T2 and T3, in contrast to the baseline time point, T1, will be included in the fixed part of the model to estimate changes from baseline to 3 months, and from baseline to 6 months. To assess the effects of interventions over time, interaction terms between the treatment group indicators, G2 and G3, and time indicators, T2 and T3, will be included in the fixed part of the model. The significantly different demographics between groups identified in the first step of the analysis and other possible confounders will be adjusted by including them in the main effect model. The within-patient random effects of the outcome measurements will naturally be taken into account by the level two variance in the model. A pairwise comparison and other hypotheses on the intervention effects can be tested using a generalised Ward test with an adequate degree of freedom from the modelling analysis. This analysis approach has two main advantages: a statistically highly efficiency and taking missing data into account. The effects of interventions on other secondary outcome measures, normally distributed and measured at three time points, such as patients’ cognitive and social functioning, experience of pleasure, and self-efficacy, will be analysed using the same model, but a separate model for each outcome. A multilevel logistic model will be used for outcomes in binary form, and multilevel multinomial models will be used for ordinal or nominal outcomes. For outcomes measured at two time points only, such as neurocognitive outcomes, the model will have only one time indicator with two interaction terms to test the efficacy of cognitive training with computerised exercises three months into the trial.

SPSS (the Statistical Package for the Social Sciences) Missing Values will be used to identify the amount and pattern of missing data. If missing values are few and randomly allocated, the last observation carried forward (LOCF) will be used to replace the missing data. If missing values are not randomly distributed, multiple imputation approaches will be used. A sensitivity analysis will be performed by comparing results from analysis datasets without missing data imputation and with imputation.

In all tests, significant differences between groups will be detected with a 95% confidence level, and *P* values of < 0.05 will be considered significant. Statistical analyses will be carried out with SAS System for Windows, version 9.4 (SAS Institute Inc.) or R statistical software (R Core Team, 2016).

#### Behavioural data analysis for neuroimaging measurement

Accuracy (%) and reaction times (ms [milliseconds]) will be recorded for the cognitive tasks [55] and additional signal detection indices will be calculated for the multi-object visual working memory task. Change in behavioural performance will be analysed for all three groups using a multilevel regression/repeated measures ANOVA, testing the task/group and by time interaction. Within this framework, group differences in change will be evaluated using contrast analyses.

##### EEG signals

For EEG signals associated with specific defined events, ERP data analysis will be conducted in Curry 8 (Compumedics, Charlotte, NC, USA). Raw continuous EEG will be re-referenced to linked mastoids and then bandpass filtered from 0.1 to 30 Hz. Eye blink artifacts will be corrected by electrooculography (EOG) regression method. Stimulus-locked epochs will be extracted from − 200 ms before to 1000 ms after the onsets of stimulus. Only trials with correct behavioural responses will be included in the subsequent ERP analyses. Epochs of the same experimental condition will be averaged to produce the ERP. The mean voltage between − 200 and 0 ms will be used for baseline correction. Electrodes chosen for analyses cover the two hemispheres and midline cortical regions. The six lateral electrodes that cover two hemispheres include Fp1 and Fp2 for prefrontal regions, F3 and F4 for the frontal regions, C3 and C4 for the central regions, T7 and T8 for the temporal regions, P3 and P4 for the parietal regions, and PO3 and PO4 for the parieto-occipital regions. The four electrodes that cover the midline regions are Fz, Cz, Pz, and POz. ERP components (P1, N1, N2 and P3), and their corresponding time windows will be identified from the grand averaged waveforms. The mean amplitude of each ERP component will be calculated for further statistical analysis.

##### MRI data analysis

The outcomes will be analysed at two points only (baseline and after 3 months). A time-series statistical analysis will be carried out using FILM (FMRIB S Improved Linear Model) with local autocorrelation correction [52]. To increase the power to detect group differences, to reduce the number of voxel-wise statistical comparisons, and to avoid risk of bias for subsequent analyses, the data from all subjects at both points of data collection (54 sessions) will be pooled to identify functional regions of interest (ROIs) for each task. Z-statistic images will be thresholded using a cluster correction method to account for multiple comparisons, as determined by Z42.3, to provide a brain-wise cluster significance threshold of p¼0.05.

### Ethical issues

The WMA Declaration of Helsinki [56] and its ethical principles for medical research involving human subjects has guided our study design and protocol. The data collection will be voluntary based; written informed consent forms will be signed by patients. Prior to enrolment, participants will be made aware of all potential risks and benefits of participation (oral and written information). All steps will be taken to avoid any harm to participants. First, to minimise extra burden due to gaming interventions, playing intensity will be set at a maximum of 5 h per week, or it will be tailored to participants’ individual needs. Second, we have chosen video games that are purely fictional and do not include intentional violence. Third, possible changes in a patient’s health status will be continually monitored throughout the project. Fourth, players in this study will play the game under supervision only. Fifth, patients’ participation may be biased toward those who are interested in gaming, which will be taken into account in the conclusions. Any extra costs to the patients as a result of participation (treatment or travel costs, gaming licenses) will be covered.

## Discussion

This study will be the first clinical trial in Hong Kong to test the effectiveness of video gaming on improving cognitive and neurocognitive functioning in people with schizophrenia. The study will offer new insights into, and characterization of, the impact of using video gaming on patients’ health and well-being. The study will also contribute towards the development of new approaches to patient care in mental health services in Hong Kong. The results may also have implications for other health conditions in which motivational problems are related to health outcomes. The topic of the study is justified by the need to develop more innovative and engaging interventions for patients with schizophrenia [13]. However, before making recommendations for patients and especially decision makers, we should have an understanding of what the clinical outcomes of gaming interventions on health and well-being are. If ICT (Information and communications technology)-based interventions are to be taken as part of routine care without a clear understanding of how they work, there is the danger of using new interventions that may be non-effective and costly. If successful, the findings of this study will have the potential for remarkable societal and clinical impact, particularly in relation to changing treatment approaches and cultures in psychiatric services. In the long run, the findings could inform whether the gaming intervention could potentially reduce service costs and if our interventions are effective. It might also be possible that the study could result in a paradigm shift for non-medicinal treatment for schizophrenia, as this type of treatment is low-cost, easy to use and effective for cognitive symptoms of schizophrenia.

## Study status

Patient recruitment is ongoing.

## Data Availability

Not applicable.

## Abbreviations

AIMS: Abnormal Movement Involuntary Scale
BARS: Barnes Akathisia Rating Scale
BSPS: Brief Social Phobia Scale
CAINS: Clinical Assessment Interview for Negative Symptoms
CDS-C: Calgary Depression Scale for Schizophrenia
DSM-IV: Diagnostic and Statistical Manual of Mental Disorders, fifth edition
EEG: Electroencephalography
ECT: Electroconvulsive therapy
EOG: Electrooculography
ERP: Event-related potential
ES: Effect size
FILM: FMRIB S Improved Linear Model
fMRI: Functional magnetic resonance imaging
GSE: General Self-Efficacy Scale
ICT: Information and communications technology
IQ: Intelligence Quotient
LNST: Letter-Number-Span-Test
LOCF: Last observation carried forward
MANOVA: Multivariate analysis of variance
MMSE: Mini Mental State Examination
MRI: Magnetic Resonance Imaging
ms: Millisecond
NGO: Non-governmental organization
NIMH-MATRICS: US National of Institute of Mental Health, the Measurement and Treatment Research to Improve Cognition in Schizophrenia
RA: Research Assistant
RCT: Randomised controlled trial
ROIs: Regions of interest
SAS: Simpson-Angus Rating Scale
SPSS: Statistical Package for the Social Sciences
TEPS: Temporal Experience of Pleasure Scale
TMT: Trail Making Test
WMS: Wechsler Memory Scale
